# Study on Precipitation and Growth of TiN in GCr15 Bearing Steel during Solidification

**DOI:** 10.3390/ma12091463

**Published:** 2019-05-06

**Authors:** Bin Li, Xiao Shi, Hanjie Guo, Jing Guo

**Affiliations:** 1 School of Metallurgical and Ecological Engineering, University of Science and Technology Beijing, Beijing 100083, China; libin4962337@163.com (B.L.); mighty_works@163.com (X.S.); guojingzq@163.com (J.G.); 2 Beijing Key Laboratory of Special Melting and Preparation of High-End Metal Materials, Beijing 100083, China

**Keywords:** GCr15 bearing steel, TiN, precipitation, growth, solidification segregation

## Abstract

In this paper, the precipitation thermodynamics and growth kinetics of TiN inclusions in GCr15 bearing steel during solidification were calculated in more detail. A more reasonable formula for calculating the segregation of the solute elements was adopted and the stability diagram of TiN precipitation considering solidification segregation was given. By solving equations, the change of the solute element content before and after TiN inclusion precipitation was calculated, and the results were substituted into the kinetic formula of the inclusion growth, which made the kinetic calculation more accurate. Results showed that the most effective way to reduce the precipitation of TiN is to increase the cooling rate and decrease the contents of Ti and N in steel. The effect of Ti content on the size of TiN inclusions is greater than that of N content.

## 1. Introduction

Bearing materials require a high fatigue life which is closely related to the purity of steel. In particular, brittle oxide inclusions and punctiform non-deformable inclusions in steel are extremely harmful to the fatigue life of the bearing material [1]. With the development of pure steel smelting technology, the cleanness of steel has been greatly improved, which gradually weakens the influence of such inclusions, while the effect of TiN inclusions with higher hardness and brittleness on the fatigue life of bearing steel is more prominent. The effect of TiN inclusions on the fatigue life of bearing steel is much greater than that of oxide inclusions of the same quantity. The impact of 6 μm nitride inclusion on fatigue performance is equivalent to that of oxides with an average size of 25 μm [2].

The precipitation of TiN in bearing steel has been studied extensively. Zhou et al. [3] and Yang et al. [4] studied the precipitation behavior of TiN during solidification, and the formation of TiN was thermodynamically calculated and experimentally analyzed by Pak et al. [5] and Fu et al. [6], who pointed out that Ti content in high quality bearing steel should be properly controlled to reduce TiN formation.

However, in these studies, the model used for calculating the segregation of solute elements does not take into account the back diffusion of solute elements, resulting in an infinite segregation rate when the solid fraction approaches 1, which is unreasonable. In addition, in the calculation of the inclusion particle growth, the change of solute element content with time has not been considered, so the integration result is doubtful.

On the basis of previous studies, the precipitation thermodynamics and growth kinetics of TiN inclusions in GCr15 bearing steel during the solidification process were calculated in more detail. A more reasonable formula for calculating the segregation of solute elements was adopted and the stability diagram of TiN precipitation considering solidification segregation was given. By solving the developed equations, the change of solute element content during the TiN inclusion precipitation was calculated. Then, the calculated results were substituted into the kinetic formula of the inclusion growth, which made the kinetic calculation more accurate. Meanwhile, the effects of Ti content, N content and cooling rate on the size of TiN are discussed, which provides a theoretical support for reducing the size of TiN inclusions in bearing steel and reducing the damage to fatigue life.

## 2. The Thermodynamics of TiN Inclusions Precipitation during Solidification

### 2.1. The Chemical Composition of GCr15 Bearing Steel and Temperature of Solidus and Liquidus Line

The chemical composition of GCr15 bearing steel is shown in Table 1. 

The liquidus temperature ($T_{L}$) and solidus temperature ($T_{S}$) of GCr15 bearing steel are calculated according to Equation (1).

(1) $$T_{L}=T_{m}-\sum \Delta T_{L}\cdot w_{B},\;T_{S}=T_{m}-\sum \Delta T_{S}\cdot w_{B}$$

where $T_{m}$ is the melting point of pure iron, 1809 K. $w_{B}$ is the mass fraction of elements in steel. $\Delta T_{L}$ or $\Delta T_{S}$ is the decrease of melting point of pure iron when the mass fraction of an element in steel increases by 1%. The values of $\Delta T_{L}$ and $\Delta T_{S}$ are shown in Table 2 [7]. 

According to the above calculation, the liquidus temperature $T_{L}$ and solidus temperature $T_{S}$ of GCr15 bearing steel are 1726 K and 1608 K, respectively. 

### 2.2. The Equilibrium Activity Product of TiN Precipitation

[Ti]+[N]=TiN(s)

(2) $$\Delta G^{⊖}=-291000+107.91T$$

As the reaction (2) reaches equilibrium state,

(3) $$\Delta G^{⊖}=-{RT\ln K}^{⊖}=-RT\ln\frac{a_{\mathrm{TiN}(s)}}{a_{\left[\mathrm{Ti}\right]}\cdot a_{\left[N\right]}}=-RT\ln\frac{1}{f_{\mathrm{Ti}}\left[\%\mathrm{Ti}\right]\cdot f_{N}\left[\%N\right]}$$

In Equation (3), $f_{\mathrm{Ti}}$ and $f_{N}$ are the activity coefficients of Ti and N at the temperature of the solidification front, respectively, which can be calculated by Equations (4) and (5) [6]:

(4) $${\mathrm{lg}f}_{\mathrm{Ti}}=(\frac{2557}{T}-0.365)\cdot {\mathrm{lg}f}_{\mathrm{Ti}}\left(1873\;K\right)$$

(5) $${\mathrm{lg}f}_{N}=(\frac{2280}{T}-0.75)\cdot {\mathrm{lg}f}_{N}\left(1873\;K\right)$$

In Equations (4) and (5), $f_{\mathrm{Ti}}\left(1873\;K\right)$ and $f_{N}\left(1873\;K\right)$ are the activity coefficients of the elements Ti and N at 1873 K, respectively.

(6) $${\mathrm{lg}f}_{\mathrm{Ti}}\left(1873\;K\right)=\sum e_{\mathrm{Ti}}^{\;j}\cdot \left(\%j\right)$$

(7) $${\mathrm{lg}f}_{N}\left(1873\;K\right)=\sum e_{N}^{\;j}\cdot (\%j)$$

where $e_{\mathrm{Ti}}^{\;j}$ and $e_{N}^{\;j}$ are the interaction coefficients of the element *j* to Ti and N in molten steel at 1873 K, respectively. The interaction coefficients used in the present study are shown in Table 3 [7]. 

By substituting the composition of bearing steel into Equations (2)–(7), the solubility product of Ti and N in molten steel at equilibrium can be obtained as follows:

(8) $$\mathrm{lg}([\%\mathrm{Ti}][\%N])=5.811-\frac{16196}{T}$$

**Figure 1:**
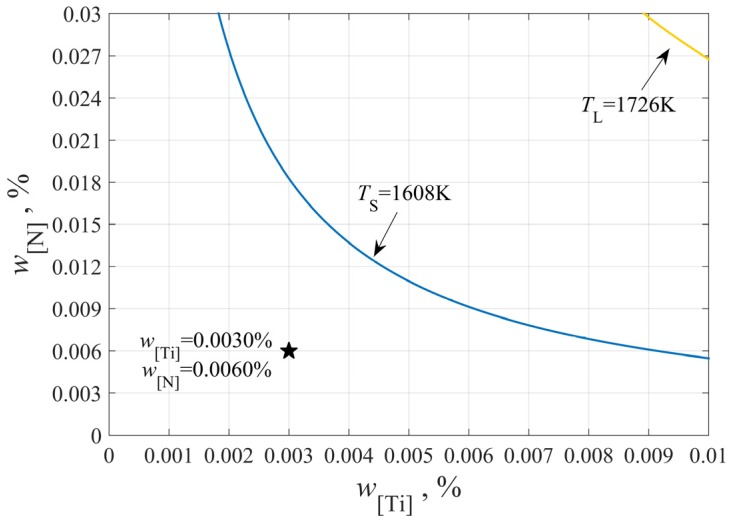
The stability diagram of TiN inclusions precipitation.

In theory, the TiN inclusions will precipitate when the actual solubility product in molten steel is larger than the equilibrium solubility product. According to Equation (8) and the solid–liquid temperature, the stability diagram of TiN precipitation can be drawn, as shown in Figure 1. 

As can be seen from Figure 1, the actual content of Ti and N in molten steel is lower than the equilibrium solubility product of TiN at the solidus temperature and much lower than that at the liquidus temperature, indicating that it is impossible for TiN to precipitate above liquidus temperature. Whether it can precipitate in the solidification process or not, it is necessary to consider the increase of solubility product caused by elements segregation. 

### 2.3. The Segregation of Solute Elements during the Solidification Process

It is defined that $C_{0}$ is the initial concentration of solute element. $C_{S}$ and $C_{L}$ are the concentrations of the solute element in the solid and liquid phase, respectively. $f_{S}$ is the volume fraction of solid. *k* is the equilibrium distribution coefficient of the solute element between the liquid and the solid phase. At equilibrium, it can be concluded from the lever law, $C_{L}=\frac{C_{0}}{\left(1-f_{S}\right)+k\cdot f_{S}}$, $C_{S}={kC}_{L}$. Scheil’s model [8] assumes that the solute elements diffuse completely in the liquid phase and non-diffuse completely in the solid phase, and Scheil’s equation $C_{L}=C_{0}{(1-f_{S})}^{k-1}$ is given. When the volume fraction of solid $f_{S}$ is close to 1, the concentrations $C_{S}$ or $C_{L}$ in Scheil’s equation will become infinite, which is obviously unrealistic. Brody and Flemings [9] assume that the solute diffuses completely in the liquid phase and partially in the solid phase, and the segregation equation is given. Clyne and Kurz [10] have revised the coefficients and proposed the C-K equation, which is widely used in most studies. On this basis, Ohnaka [11] has proposed a more elegant approximation to the microsegregation problem with back diffusion. Finally, Kobayashi [12] has developed an exact analytical solution to the microsegregation problem and has also provided some higher order approximations, as shown in Equation (9):

(9) $$C_{L}=C_{0}\xi^{\eta }\left\{1+U\left[\frac{1}{2}\left(\frac{1}{\xi^{2}}-1\right)-2\left(\frac{1}{\xi }-1\right)-\ln\xi \right]\right\}$$

$$\mathrm{With}\;\xi =1-(1-{\beta k)f}_{S}$$

$$\beta =\frac{2\gamma }{1+2\gamma }$$

$$\gamma =\frac{{8D}_{S}t_{f}}{L^{2}}$$

$$\eta =\frac{k-1}{1-\beta k}$$

$$U=\frac{\beta^{3}k(k-1)[(1+\beta )k-2]}{4\gamma {\left(1-\beta k\right)}^{3}}$$

where $D_{S}$ is the diffusion coefficient of the solute element in the solid phase. $t_{f}$ is the local solidification time, which is calculated by the formula $t_{f}=\frac{T_{L}-T_{S}}{R_{C}}$. $R_{C}$ is the cooling rate and the value is set to 5 K/s. *L* is the secondary dendrite arm spacing, which can be expressed as a function of cooling rate and generally calculated by the formula $L=146\times 10^{-6}R_{C}^{-0.39}$. 

Moreover, the temperature in front of the solid–liquid interface during solidification can be expressed by the following formula:

(10) $$T=T_{m}-\frac{T_{m}-T_{L}}{1-f_{S}\times \frac{T_{L}-T_{S}}{T_{m}-T_{S}}}$$

**Figure 2:**
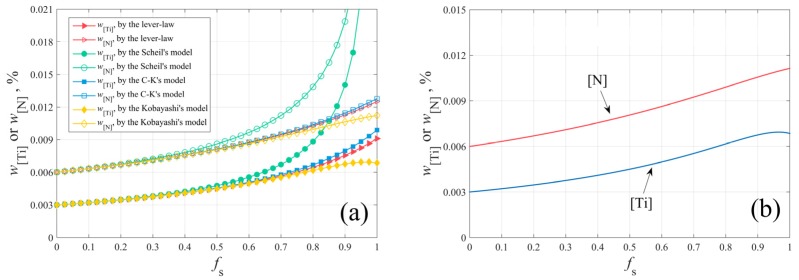
The solidification segregation of Ti and N in GCr15 bearing steel: (**a**) based on the different segregation models; (**b**) based on the segregation model by Kobayashi.

The segregation of Ti and N during solidification can be calculated, based on the different segregation models, as shown in Figure 2a. The lever law describes the solidification process at equilibrium. Scheil’s model is an extreme case, and it assumes that solute elements diffuse completely in the liquid phase and non-diffuse completely in the solid phase. The widely used C-K model has not considered the back diffusion of solute elements in the solid phase. Therefore, the more reasonable model proposed by Kobayashi [13] was adopted and its results were verified by previous studies. The segregation of Ti and N during solidification based on the segregation Equation (9) is illustrated in Figure 2b. 

### 2.4. The Stability Diagram of TiN Inclusions Precipitation Considering Solidification Segregation

**Figure 3:**
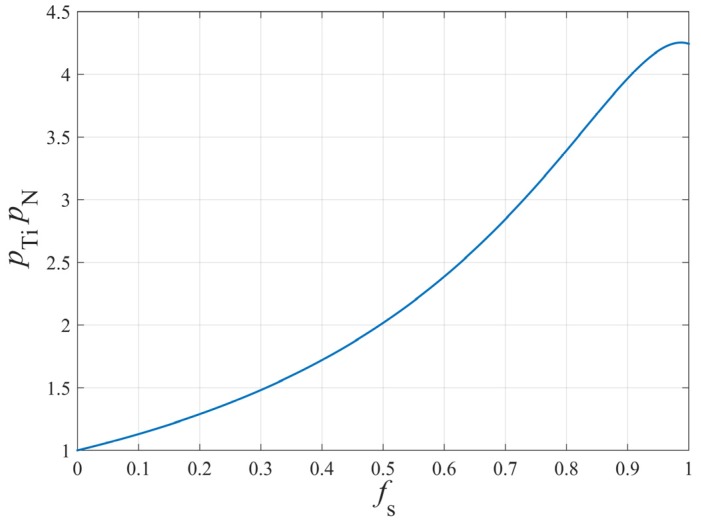
The relationship between $p_{\mathrm{Ti}}p_{N}$ and $f_{S}$.

Due to the segregation of solute elements, such as Ti and N, during solidification, TiN will still precipitate even if the initial solubility product of Ti and N in molten steel is lower than the equilibrium solubility product of TiN at solidus temperature. By defining the segregation ratio of element $p=\frac{C_{L}}{C_{0}}$, and considering solidification segregation, the actual solubility product in molten steel is $Q^{\prime }={\left[\%\mathrm{Ti}\right]}_{L}{\left[\%N\right]}_{L}=p_{\mathrm{Ti}}p_{N}{\left[\%\mathrm{Ti}\right]}_{0}{\left[\%N\right]}_{0}$. The relationship between the product of the segregation ratio of Ti and N ($p_{\mathrm{Ti}}p_{N}$) and the volume fraction of solid ($f_{S}$) is shown in Figure 3. 

It can be seen from Figure 3 that the actual concentration $Q^{\prime }$ of Ti and N in molten steel has a maximum when solidification is proceeding. If the actual solubility product is always less than the equilibrium solubility product during solidification, TiN will not precipitate; on the contrary, TiN will precipitate during solidification. From Equation (8), it can be derived that:

(11) $$\mathrm{lg}\left({\left([\%\mathrm{Ti}][\%N]\right)}_{\max}\right)=\mathrm{lg}\left({{(p}_{\mathrm{Ti}}p_{N})}_{\max}\left[\%\mathrm{Ti}][\%N\right]\right)=5.811-\frac{16196}{T}$$

**Figure 4:**
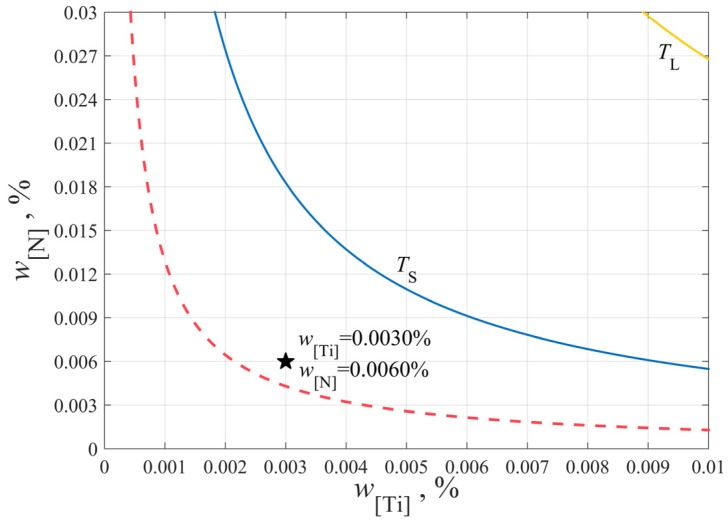
The stability diagram of TiN inclusions precipitation considering solidification segregation.

Based on the above calculation, the stability diagram of TiN inclusions precipitation considering solidification segregation is given, as shown in Figure 4. 

The initial composition of Ti and N in molten steel is [%Ti]=0.0030, [%N]=0.0060, respectively. Although the initial solubility product is lower than the equilibrium solubility product of TiN at solidus temperature, the actual solubility product is higher than the equilibrium solubility product due to the segregation of elements, giving rise to the fact that the TiN will still precipitate during solidification. The red dotted line in Figure 4 indicates that unless the Ti and N contents in molten steel are controlled in the area below the red line, TiN inclusions will precipitate during solidification. That is to say, the contents of Ti and N on the red line are the critical values that determine whether TiN can precipitate during solidification or not. Therefore, in the production of bearing steel, the contents of Ti and N elements in molten steel must be well controlled in order to avoid the formation of TiN inclusions in the liquid and solid–liquid phases.

### 2.5. The Precipitation of TiN Inclusions during Solidification

**Figure 5:**
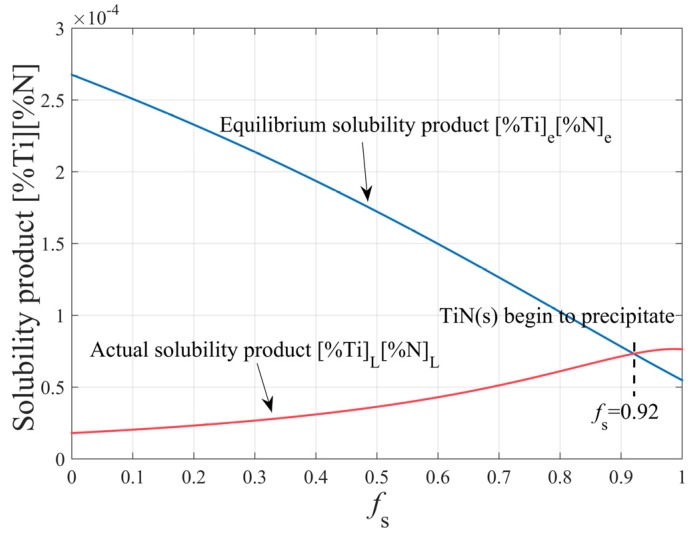
The thermodynamic conditions of TiN precipitation during solidification.

As solidification proceeds, the contents of Ti and N in molten steel increase gradually due to segregation. When the actual solubility product in molten steel is more than the equilibrium solubility product, the TiN inclusions will precipitate. According to the initial composition of molten steel and the above calculation, the TiN inclusions begin to precipitate when the volume fraction of solid ($f_{S}$) is greater than 0.92, as shown in Figure 5. 

The mass of the Ti and N elements in the TiN inclusions must meet the stoichiometric ratio, that is, the mass ratio of the lessened Ti and N elements in the liquid phase is 48/14. In addition, following the precipitation of TiN, the contents of Ti and N remaining in the liquid must meet the equation of the solubility product at equilibrium.

[Ti]+[N]=TiN(s)

(12) $${\mathrm{lg}([\%\mathrm{Ti}]}_{e}\cdot {\left[\%N\right]}_{e})=5.811-\frac{16196}{T}$$

(13) $$\frac{{\left[\%\mathrm{Ti}\right]}_{L}-{\left[\%\mathrm{Ti}\right]}_{e}}{{\left[\%N\right]}_{L}-{\left[\%N\right]}_{e}}=\frac{M_{\mathrm{Ti}}}{M_{N}}=\frac{48}{14}$$

**Figure 6:**
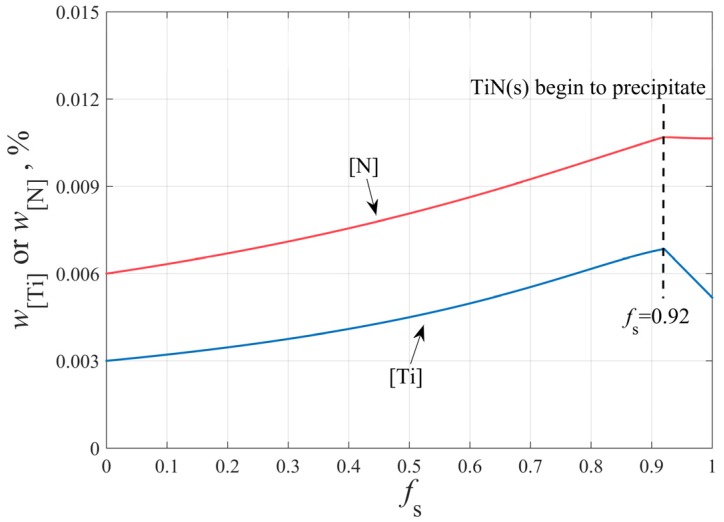
The contents of Ti and N in molten steel during solidification.

${\left[\%\mathrm{Ti}\right]}_{L}$ and ${\left[\%N\right]}_{L}$ in Equation (13) are given by the segregation Equation (9), then ${\left[\%\mathrm{Ti}\right]}_{e}$ and ${\left[\%N\right]}_{e}$ are obtained by solving simultaneous Equations (12) and (13). Given the above, the contents of Ti and N in molten steel during solidification are shown in Figure 6. 

## 3. The Growth of TiN Inclusions during Solidification

### 3.1. The Basic Equation of TiN Inclusions’ Growth Dynamics

According to the comparison of the diffusion coefficient of N in molten steel ($D_{L–N}=3.25\times 10^{-7}e^{\frac{-11500}{RT}},\;m^{2}/s$, [7]) and that of Ti ($D_{L–\mathrm{Ti}}=3.1\times 10^{-7}e^{\frac{-11500}{RT}},\;m^{2}/s$, [13]), the diffusion of Ti is slower than that of N in liquid steel and the enrichment of Ti is easier than that of N in the solidification front. So, the solute element N which spreads faster is a restrictive factor upon the growth of TiN inclusions. Based on Fick’s first law, the dynamic formula of TiN inclusions’ growth is derived. For the formula of inclusions’ growth, Hong and DebRoy [14,15] proposed a formula which is similar in form to the formula in this study, though with less detail of subsequent calculations. The formula used by Goto et al. [13,16] is almost the same as that used in this study, but it seems to be incorrect in form.

**Figure 7:**
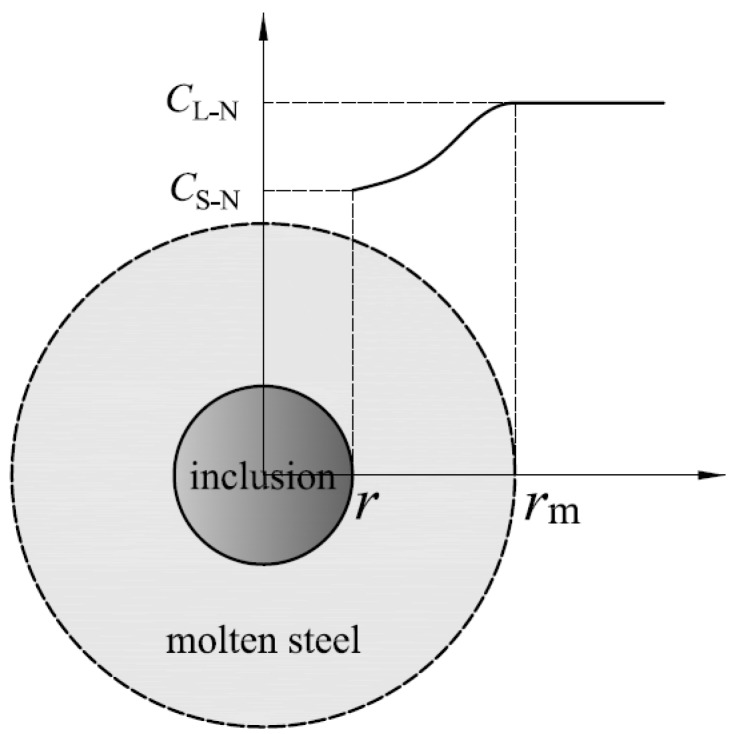
The diagrammatic sketch of N element diffusion in molten steel.

As shown in Figure 7, $C_{L–N}$ is the concentration of N in molten steel; $C_{S–N}$ is the concentration of N at the inclusion–molten steel interface; $C_{e–N}$ is the concentration of N at equilibrium; $r_{m}$ is the radius of imaginary molten steel balls and *r* is the radius of TiN inclusions. 

According to Fick’s First Law, Equation (14) can be obtained

(14) $$-\frac{{dn}_{N}}{dt}={4\pi r}^{2}D_{L–N}\frac{{dc}_{N}}{dr}$$

Integrating Equation (14),

(15) $$\int_{C_{L–N}}^{C_{S–N}}{dc}_{N}=-\frac{1}{{4\pi D}_{L–N}}\frac{{dn}_{N}}{dt}\int_{r_{m}}^{r}\frac{dr}{r^{2}}$$

(16) $$-\frac{{dn}_{N}}{dt}={4\pi D}_{L–N}\frac{r_{m}r}{r_{m}-r}{(C}_{L–N}-C_{S–N})$$

Since the controlling step of TiN growth is the diffusion of N in molten steel, the concentration of N at the inclusion–molten steel interface is equal to that at equilibrium, i.e., $C_{S–N}=C_{e–N}$.

(17) $$-\frac{{dn}_{N}}{dt}=\frac{{dn}_{\mathrm{TiN}}}{dt}=\frac{{dm}_{\mathrm{TiN}}}{M_{\mathrm{TiN}}dt}=\frac{\rho_{\mathrm{TiN}}}{M_{\mathrm{TiN}}}\frac{d\left(\frac{4}{3}{\pi r}^{3}\right)}{dt}=\frac{{4\pi r}^{2}\rho_{\mathrm{TiN}}}{M_{\mathrm{TiN}}}\frac{dr}{dt}$$

Obtained by Equation (17),

(18) $$\frac{{4\pi r}^{2}\rho_{\mathrm{TiN}}}{M_{\mathrm{TiN}}}\frac{dr}{dt}={4\pi D}_{L–N}\frac{r_{m}r}{r_{m}-r}{(C}_{L–N}-C_{S–N})={4\pi D}_{L–N}\frac{r_{m}r}{r_{m}-r}{(C}_{L–N}-C_{e–N})$$

Assuming that $r_{m}$ is infinite, then,

(19) $$\frac{{4\pi r}^{2}\rho_{\mathrm{TiN}}}{M_{\mathrm{TiN}}}\frac{dr}{dt}={4\pi D}_{L–N}\frac{r}{1-\frac{r}{r_{m}}}{(C}_{L–N}-C_{e–N})={4\pi rD}_{L–N}{(C}_{L–N}-C_{e–N})$$

That is,

(20) $$r\frac{dr}{dt}=D_{L–N}\frac{M_{\mathrm{TiN}}}{\rho_{\mathrm{TiN}}}{(C}_{L–N}-C_{e–N})$$

Obviously,

(21) $$c_{N}=\frac{n_{N}}{V_{\mathrm{metal}}}=\frac{m_{N}}{M_{N}\frac{m_{\mathrm{metal}}}{\rho_{\mathrm{metal}}}}=\frac{\rho_{\mathrm{metal}}}{M_{N}}\frac{m_{N}}{m_{\mathrm{metal}}}=\frac{\rho_{\mathrm{metal}}}{M_{N}}\frac{\left[\%N\right]}{100}$$

Thus,

(22) $$r\frac{dr}{dt}=D_{L–N}\frac{M_{\mathrm{TiN}}}{\rho_{\mathrm{TiN}}}\frac{\rho_{\mathrm{metal}}}{M_{N}}(\frac{{\left[\%N\right]}_{L}}{100}-\frac{{\left[\%N\right]}_{e}}{100})=\frac{D_{L–N}}{100}\frac{M_{\mathrm{TiN}}}{M_{N}}\frac{\rho_{\mathrm{metal}}}{\rho_{\mathrm{TiN}}}({\left[\%N\right]}_{L}-{\left[\%N\right]}_{e})$$

In the above equations, $n_{N}$ is the mole number of N element and $n_{\mathrm{TiN}}$ is the mole number of TiN inclusion; $m_{N}$ is the mass of N element and $m_{\mathrm{TiN}}$ is the mass of TiN inclusion; $c_{N}$ is the concentration of N element; *t* is the growth time of inclusion particles; ${\left[\%N\right]}_{L}$ is the mass percent concentration of N in molten steel, and ${\left[\%N\right]}_{e}$ is the mass percent concentration of N at equilibrium; $D_{L–N}$ is the diffusion coefficient of N in molten steel; $M_{N}$ is the atomic weight of N and $M_{\mathrm{TiN}}$ is the molecular weight of TiN, respectively; $\rho_{\mathrm{metal}}$ is the density of liquid steel and $\rho_{\mathrm{TiN}}$ is the density of TiN, valued $\rho_{\mathrm{metal}}=7070\;\mathrm{kg}/m^{3}$ and $\rho_{\mathrm{TiN}}=5430\;\mathrm{kg}/m^{3}$, respectively. 

### 3.2. The Maximum Size of TiN Inclusion

**Figure 8:**
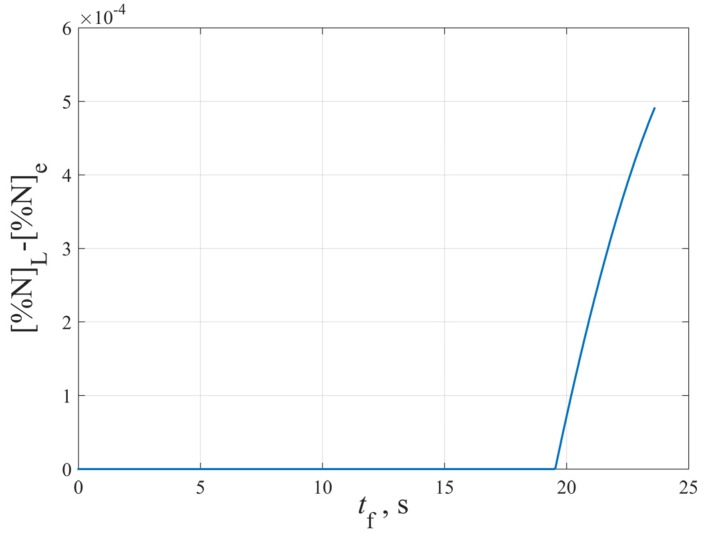
The relationship between ${\left[\%N\right]}_{L}-{\left[\%N\right]}_{e}$ and the solidification time $t_{f}$.

Due to the segregation of solute elements during solidification, ${\left[\%N\right]}_{L}$ in Equation (22) varies with the solidification time $t_{f}$ or the inclusion growth time *t*, so ${\left[\%N\right]}_{L}-{\left[\%N\right]}_{e}$ should not be treated as a constant when integrating Equation (22), as several researchers have done [4,17,18]. According to the results obtained by solving the equations in Section 2.5, the relationship between ${\left[\%N\right]}_{L}-{\left[\%N\right]}_{e}$ and the solidification time $t_{f}$ can be obtained, as shown in Figure 8. 

As can be seen from Figure 8, starting from the precipitation of TiN inclusions, ${\left[\%N\right]}_{L}-{\left[\%N\right]}_{e}$ is approximately linear with the inclusion growth time *t*, so let ${\left[\%N\right]}_{L}-{\left[\%N\right]}_{e}=bt$, where *b* is a constant independent of *t*.

Then,

(23) $$r\frac{dr}{dt}=\frac{D_{L–N}}{100}\frac{M_{\mathrm{TiN}}}{M_{N}}\frac{\rho_{\mathrm{metal}}}{\rho_{\mathrm{TiN}}}({\left[\%N\right]}_{L}-{\left[\%N\right]}_{e})=\frac{D_{L–N}}{100}\frac{M_{\mathrm{TiN}}}{M_{N}}\frac{\rho_{\mathrm{metal}}}{\rho_{\mathrm{TiN}}}bt$$

Separating variables and integrating,

(24) $$\int_{r_{0}}^{r_{t}}rdr=\frac{{bD}_{L–N}}{100}\frac{M_{\mathrm{TiN}}}{M_{N}}\frac{\rho_{\mathrm{metal}}}{\rho_{\mathrm{TiN}}}\int_{0}^{t}tdt$$

(25) $$\frac{r_{t}^{2}}{2}-\frac{r_{0}^{2}}{2}=\frac{{bD}_{L–N}}{100}\frac{M_{\mathrm{TiN}}}{M_{N}}\frac{\rho_{\mathrm{metal}}}{\rho_{\mathrm{TiN}}}\frac{t^{2}}{2}$$

(26) $$r_{t}^{2}=\frac{{bD}_{L–N}}{100}\frac{M_{\mathrm{TiN}}}{M_{N}}\frac{\rho_{\mathrm{metal}}}{\rho_{\mathrm{TiN}}}t^{2}+r_{0}^{2}$$

(27) $$r_{t}=\sqrt{\frac{{bD}_{L–N}}{100}\frac{M_{\mathrm{TiN}}}{M_{N}}\frac{\rho_{\mathrm{metal}}}{\rho_{\mathrm{TiN}}}t^{2}+r_{0}^{2}}$$

where $r_{0}$ is the initial radius of the inclusion particle, and $r_{t}$ is the radius of the inclusion particle at time *t*. 

**Figure 9:**
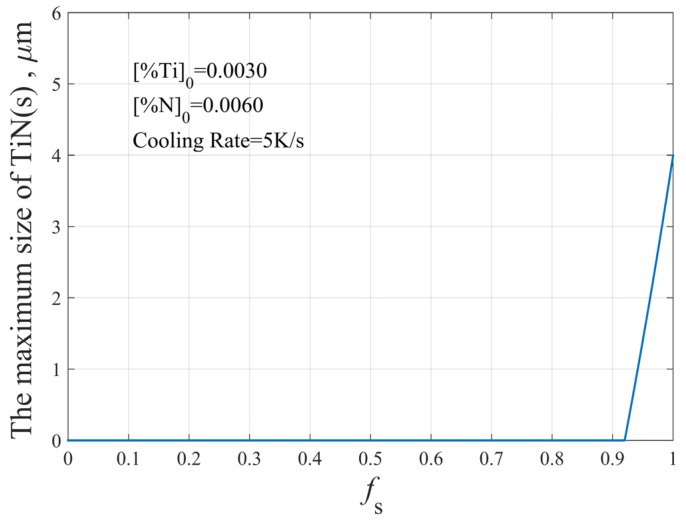
The theoretical maximum size of TiN inclusions at the end of solidification.

According to the above calculation, the theoretical maximum size of TiN inclusions at the end of solidification is obtained, as shown in Figure 9. Although the initial radius of the inclusion particle $r_{0}$ is included in Equation (27), it is an integral result. According to the calculation in this study, TiN inclusions are formed during solidification. The criterion for judging whether TiN precipitates is that the actual solubility product is larger than the equilibrium solubility product, so the initial radius $r_{0}$ is set to 0. It is possible that TiN inclusions increase with oxides or other types of inclusions as a core, but the growth of composite inclusions is complex and beyond the scope of this study.

### 3.3. The Effect of Cooling Rate on the Maximum Size of TiN Inclusions

**Figure 10:**
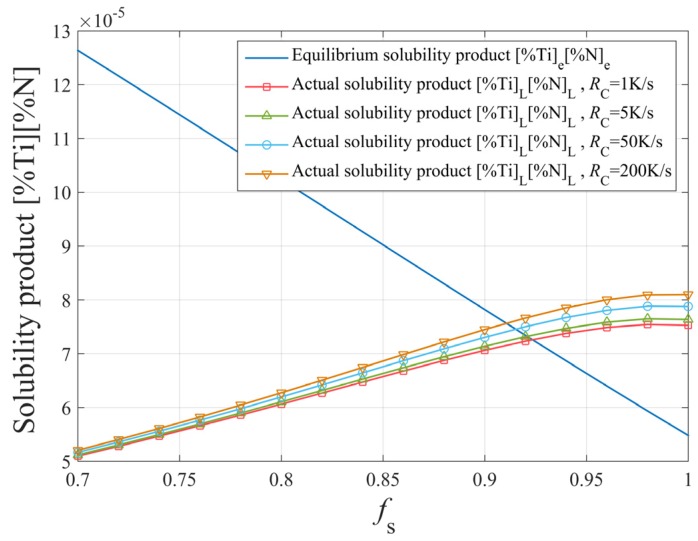
The effect of cooling rate on the segregation of solute elements Ti and N.

The effect of cooling rate on the segregation of the solute elements Ti and N is shown in Figure 10. The cooling rate has little effect on the segregation, while it affects the solidification time. The larger the cooling rate, the shorter the solidification time, which affects the maximum size of TiN inclusions. 

**Figure 11:**
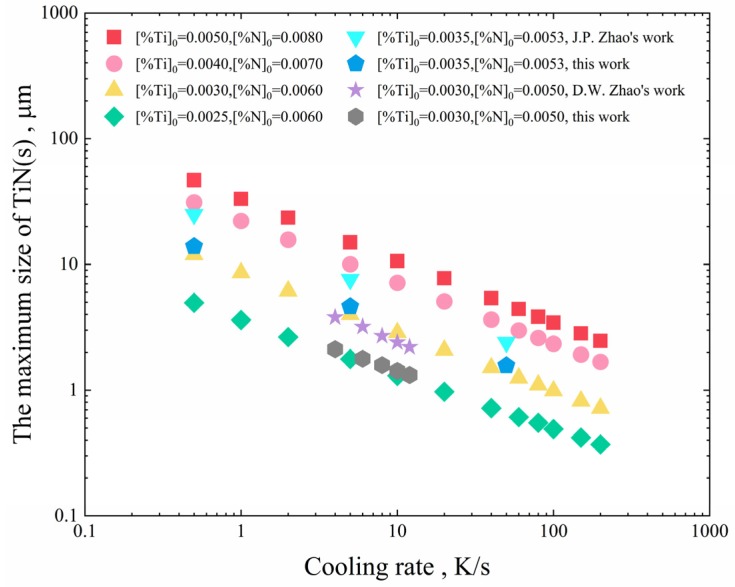
The effect of cooling rate on the maximum size of TiN inclusions.

The effect of cooling rate on the maximum size of TiN inclusions with different initial Ti and N contents is shown in Figure 11. The higher the cooling rate, the smaller the maximum size of precipitated TiN inclusions. However, when the cooling rate is sufficiently high, the maximum size of TiN inclusions is less affected by the cooling rate. In order to avoid the formation of large-sized TiN inclusions, the cooling rate should be controlled to at least 20 K/s. 

Zhao [19] calculated the maximum size of TiN inclusions when ${\left[\%\mathrm{Ti}\right]}_{0}=0.0035$, ${\left[\%N\right]}_{0}=0.0053$, and the cooling rate was 0.5 K/s, 5 K/s and 50 K/s, respectively. Zhao [20] calculated the maximum size of TiN inclusions when ${\left[\%\mathrm{Ti}\right]}_{0}=0.0030$, ${\left[\%N\right]}_{0}=0.0050$, and the cooling rate was 4 K/s, 6 K/s, 8 K/s, 10 K/s, and 12 K/s, respectively. In this study, the maximum size of TiN inclusions was calculated based on the data reported by Zhao [19] and Zhao [20]. As can be seen from Figure 11, the results of this study are lower than those calculated by Zhao [19] and Zhao [20]. The reason is that different segregation models are used, which have a great influence on the content of Ti and N in molten steel. Using Scheil’s model or the C-K model will result in a larger segregation value. Regardless of the segregation model used, for the same segregation model, the size of TiN inclusions has little relationship with the segregation of the solute elements Ti and N. However, different segregation models result in different contents of Ti and N elements in molten steel, which affect the size of inclusions in two aspects. Firstly, different contents of Ti and N elements lead to a difference in the time when TiN begins to precipitate. The higher the concentration of the solute elements, the longer the time of inclusions’ growth. Secondly, different segregation models lead to different ${\left[\%N\right]}_{L}-{\left[\%N\right]}_{e}$ in Equation (22). The larger the concentration gradient, the larger the size of inclusions. 

### 3.4. The Effect of Ti and N Contents in Molten Steel on the Maximum Size of TiN Inclusions

**Figure 12:**
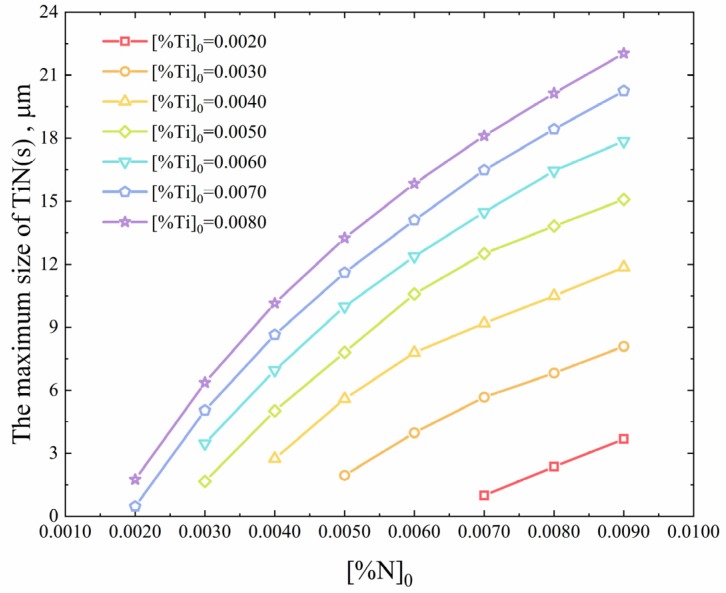
The maximum size of TiN inclusions at different initial Ti and N contents.

The initial contents of Ti and N in molten steel have a decisive influence on the precipitation and growth of TiN. The straightforward way to reduce the size of precipitated TiN inclusions is to reduce the Ti and N content in molten steel. The maximum size of precipitated TiN inclusions at different initial Ti and N contents is shown in Figure 12. When the N content is constant, reducing the Ti content in molten steel can effectively reduce the precipitation of TiN inclusions and the size of TiN inclusions. In particular, when the Ti content is low, the effect is more significant. Even when the content of N is 70 ppm and the content of Ti decreases from 30 ppm to 20 ppm, the maximum size of TiN inclusion decreases from 5.7 μm to 1.0 μm. In contrast, when Ti content is constant, the maximum size of TiN inclusions can be uniformly reduced by decreasing N content in steel. For every 10 ppm decrease in N content in steel, the maximum size of TiN can be reduced by approximately 2 μm. Therefore, it is concluded that the effect of Ti content on the size of TiN inclusions is greater than that of N content on the size of TiN inclusions. 

In the steelmaking process, the key to controlling the N element is the strength of the vacuum treatment and the result of the atmosphere protection during casting. In general, it is difficult to reduce the N content in steel to less than 50 ppm. The key to controlling the Ti element lies in the usage of different quality ferroalloys. Titanium is an element that cannot be readily removed in the steelmaking process. In the smelting of bearing steel, the main source of titanium is ferrochromium containing titanium. The content of titanium in different grades of ferrochromium varies greatly, about 0.01%–0.5%. The use of high-quality ferroalloys with low Ti content has a direct effect on reducing the Ti element in steel, but it will bring about an increase in cost. In order to produce high-quality bearing steel, the contents of Ti and N must be strictly controlled to avoid the precipitation of TiN during solidification and to maintain the precipitated TiN inclusions at a small size so as to reduce the adverse effect on the fatigue life of bearing steel. 

## 4. Conclusions

1. The precipitation model of TiN in GCr15 bearing steel during solidification was established. At the solidification front, as the solid fraction increases, the Ti and N elements will segregate. When the thermodynamic conditions of TiN inclusion formation are satisfied, the TiN inclusions will precipitate in the solid–liquid zone;
2. Before the precipitation of TiN inclusions, the contents of Ti and N increase continuously with the increase of the solid fraction. After the precipitation of TiN inclusions in liquid, the contents of Ti and N decrease with the increase of solid fraction;
3. The cooling rate of molten steel has no significant effect on the segregation of Ti and N elements at the solidification front, but it has a significant effect on the size of precipitated TiN. As the cooling rate increases, the growth time of TiN inclusions decreases, and the size of TiN inclusions decreases accordingly;
4. The most effective way to reduce the precipitation of TiN is to increase the cooling rate and decrease the contents of Ti and N in steel. The effect of Ti content on the size of TiN inclusions is greater than that of N content.

## Figures and Tables

**Table 1:** The chemical composition of GCr15 bearing steel (wt %).

C	Si	Mn	P	S	Cr	Ti	N
0.99	0.24	0.30	0.011	0.003	1.45	0.0030	0.0060

**Table 2:** The values of $\;\Delta T_{L}$ and $\Delta T_{S}$ (°C).

Elements	C	Si	Mn	P	S	Cr	Ti	N
Liquidus $\;\Delta T_{L}$	78	7.6	4.9	34.4	38.0	1.3	20	90.0
Solidus $\Delta T_{S}$	184.3	40.8	8.6	76.7	76.7	3.4	40	—

**Table 3:** The interaction coefficients of element *j* to Ti and N at 1873 K.

Element *j*	C	Si	Mn	P	S	Cr	Ti	N
$e_{\mathrm{Ti}}^{\;j}(1873\;K$)	−0.19	2.1	−0.043	−0.06	−0.27	0.022	0.042	−2.06
$e_{N}^{\;j}(1873\;K$)	0.14	0.048	−0.02	0.059	0.007	−0.046	−0.6	0
